# Salivary DNA Methylation as an Epigenetic Biomarker for Head and Neck Cancer. Part II: A Cancer Risk Meta-Analysis

**DOI:** 10.3390/jpm11070606

**Published:** 2021-06-26

**Authors:** Óscar Rapado-González, Cristina Martínez-Reglero, Ángel Salgado-Barreira, María Arminda Santos, Rafael López-López, Ángel Díaz-Lagares, María Mercedes Suárez-Cunqueiro

**Affiliations:** 1 Department of Surgery and Medical-Surgical Specialties, Medicine and Dentistry School, Universidade de Santiago de Compostela, 15782 Santiago de Compostela, Spain; oscar.rapado@rai.usc.es (Ó.R.-G.); maría.santos@iucs.cespu.pt (M.A.S.); 2 Translational Medical Oncology Group (Oncomet), Liquid Biopsy Analysis Unit, Health Research Institute of Santiago (IDIS), 15706 Santiago de Compostela, Spain; 3 Centro de Investigación Biomédica en Red de Cáncer (CIBERONC), Instituto de Salud Carlos III, 28029 Madrid, Spain; rafa.lopez.lopez@gmail.com (R.L.-L.); angel.diaz.lagares@sergas.es (Á.D.-L.); 4 Methodology and Statistics Unit, Galicia Sur Health Research Institute (IISGS), 36312 Vigo, Spain; cristina.martinez@iisgaliciasur.es (C.M.-R.); angel.salgado.barreira@sergas.es (Á.S.-B.); 5 Department of Oral Rehabilitation, Instituto Universitario de Ciências da Saúde (IUCS), 1317|4585-116 Gandra, Portugal; 6 Translational Medical Oncology Group (Oncomet), Health Research Institute of Santiago (IDIS), Complexo Hospitalario Universitario de Santiago de Compostela (SERGAS), 15706 Santiago de Compostela, Spain; 7 Cancer Epigenomics, Translational Medical Oncology Group (Oncomet), Health Research Institute of Santiago (IDIS), University Clinical Hospital of Santiago (CHUS/SERGAS), 15706 Santiago de Compostela, Spain

**Keywords:** DNA methylation, epigenetics, head and neck cancer, saliva, biomarkers, liquid biopsy, meta-analysis

## Abstract

Aberrant methylation of tumor suppressor genes has been reported as an important epigenetic silencer in head and neck cancer (HNC) pathogenesis. Here, we performed a comprehensive meta-analysis to evaluate the overall and specific impact of salivary gene promoter methylation on HNC risk. The methodological quality was assessed using the Newcastle–Ottawa scale (NOS). Odds ratios (ORs) and 95% confidence intervals (CIs) were calculated to evaluate the strength of the association and Egger’s and Begg’s tests were applied to detect publication bias. The frequency of salivary DNA promoter methylation was significantly higher in HNC patients than in healthy controls (OR: 8.34 (95% CI = 6.10–11.39; *p* < 0.01). The pooled ORs showed a significant association between specific tumor-related genes and HNC risk: *p16* (3.75; 95% CI = 2.51–5.60), *MGMT* (5.72; 95% CI = 3.00–10.91), *DAPK* (5.34; 95% CI = 2.18–13.10), *TIMP3* (3.42; 95% CI = 1.99–5.88), and *RASSF1A* (7.69; 95% CI = 3.88–15.23). Overall, our meta-analysis provides precise evidence on the association between salivary DNA promoter hypermethylation and HNC risk. Thus, detection of promoter DNA methylation in saliva is a potential biomarker for predicting HNC risk.

## 1. Introduction

The important role of epigenetic mechanisms in carcinogenesis has been widely reported. Identification of specific genes that are altered by aberrant epigenetic processes contributes to better understanding molecular pathogenesis in HNC [1]. As one of the most important epigenetic alterations, DNA hypermethylation may lead to transcriptional silencing of tumor suppressor genes and, thus, interfere in signaling pathways that control vital cell processes, such as DNA repair, apoptosis, cell proliferation, and cell-to-cell adhesion [2]. Gene promoter methylation is a common epigenetic event in early carcinogenesis, and therefore represents a promising biomarker for high-risk group stratification, early cancer detection, and prognosis prediction [3]. Numerous studies have evaluated DNA methylation as a biomarker in a wide variety of tumors [4,5,6,7]. Hypermethylation of tumor-related genes, such as cyclin-dependent kinase inhibitor 2A (*CDKN2A*), E-cadherin (*CDH1*), death-associated protein kinase (*DAPK*), phosphatase and tensin homolog (*PTEN*), and O6-methylguanine-DNA methyltransferase (*MGMT*), have been reported in HNC [8]. Likewise, various studies have focused on the detection of DNA methylation in liquid biopsies in HNC [9,10,11]. Although evidence suggests a potential association between aberrant salivary DNA methylation patterns and HNC risk, no prior research assessing overall impact is available. Therefore, we conducted a systematic review and meta-analysis to gain better insight into the magnitude of the association between salivary DNA hypermethylation and HNC risk.

## 2. Materials and Methods

### 2.1. Protocol and Registration

This study was conducted according to Preferred Reporting Items for Systematic Reviews and Meta-analysis (PRISMA) guidelines [12], and the protocol was registered with the International Prospective Register of Systematic Reviews (reference No. CRD42020199123).

### 2.2. Search Strategy, Study Selection, and Data Extraction

The search strategy and data extraction were previously described in Part I [13].

### 2.3. Selection Criteria

The inclusion criteria were as follows: (1) case-control studies; (2) studies based on salivary DNA hypermethylation biomarkers for HNC; and (3) sufficient data to calculate odds ratios (ORs) and corresponding 95% confidential intervals (CIs). The exclusion criteria were as follows: (1) reviews, letters, personal opinions, book chapters, case reports, conference abstracts, and meetings; (2) duplicate publications; (3) incomplete data; and (4) in vitro or in vivo animal experiments. 

### 2.4. Assessment of Study Quality

Independent investigators evaluated methodological quality by applying the Newcastle–Ottawa scale (NOS) [14] to each study selected. Discrepancies were resolved by consensus. For the interpretation of meta-analytic data, the NOS scale was used to score the quality of non-randomized studies based on their design, content, and ease of use. Items were scored according to a “star system” and fell under three broad categories: study group selection, group comparability, and ascertainment of exposure/outcome for case-control or cohort studies. The maximum quality score for each item was one star, except for the comparability item, which had a maximum of two stars. The NOS score ranged from 0 to 9 stars, with 8–9 stars being high quality; 6–7 stars being medium quality; and <5 stars being low quality.

### 2.5. Statistical Analysis

Statistical analysis was conducted using the meta package of free R software (v.3.4.4; https://www.r-project.org, accessed 30 November 2020). The pooled odds ratios (ORs) and their 95% confidence intervals (CIs) were calculated to assess the strength of the association between salivary promoter methylation and HNC. To evaluate the statistical model applied to the meta analytic database, heterogeneity was assessed on the basis of *I*-square (I^2^) value and Cochran’s Q statistic test-based Chi-squared test. Heterogeneity was considered significant when I^2^ > 50% and/or presence of a *p* < 0.10 for the Cochran’s Q test. If significant heterogeneity was detected, the DerSimonian and Laird random-effects model was applied to calculate the pooled OR with 95% CIs; otherwise, the Mantel–Haenszel fixed-effects model was used. Meta-regression and subgroup analyses were performed to explore the potential sources of heterogeneity among studies insofar as anatomic tumor location, sample type, sample size, DNA methylation method, and methylation gene profiling. Publication bias was assessed by Begg’s and Egger’s tests, and funnel plot inspection [15,16]. Begg’s rank test examines the correlation between the effect sizes and their corresponding sampling variances. Egger’s test regresses the standardized effect sizes on their precisions. In the presence of publication bias, both tests will be statistically significant. Moreover, publication bias was based on visual funnel-plot inspection, which shows the relationship between individual log ORs and their standard errors. The asymmetry of the funnel plot could indicate publication bias. 

*p* < 0.05 was considered to be statistically significant.

## 3. Results

### 3.1. Study Selection and Characteristics of Included Studies

The main characteristics of the included studies have already been described in Part I [13].

### 3.2. Study Quality

Bias risk and quality were assessed according to NOS (Table S1). With respect to the selection category, each of the included studies was considered adequate. Regarding comparability, 5 out of the remaining 18 studies matched for age or gender, and 2 studies matched for at least one additional risk factor. Therefore, the median NOS score in our meta-analysis was 7.33 stars.

### 3.3. Association between Salivary DNA Promoter Hypermethylation and HNC Risk

**Figure 1:**
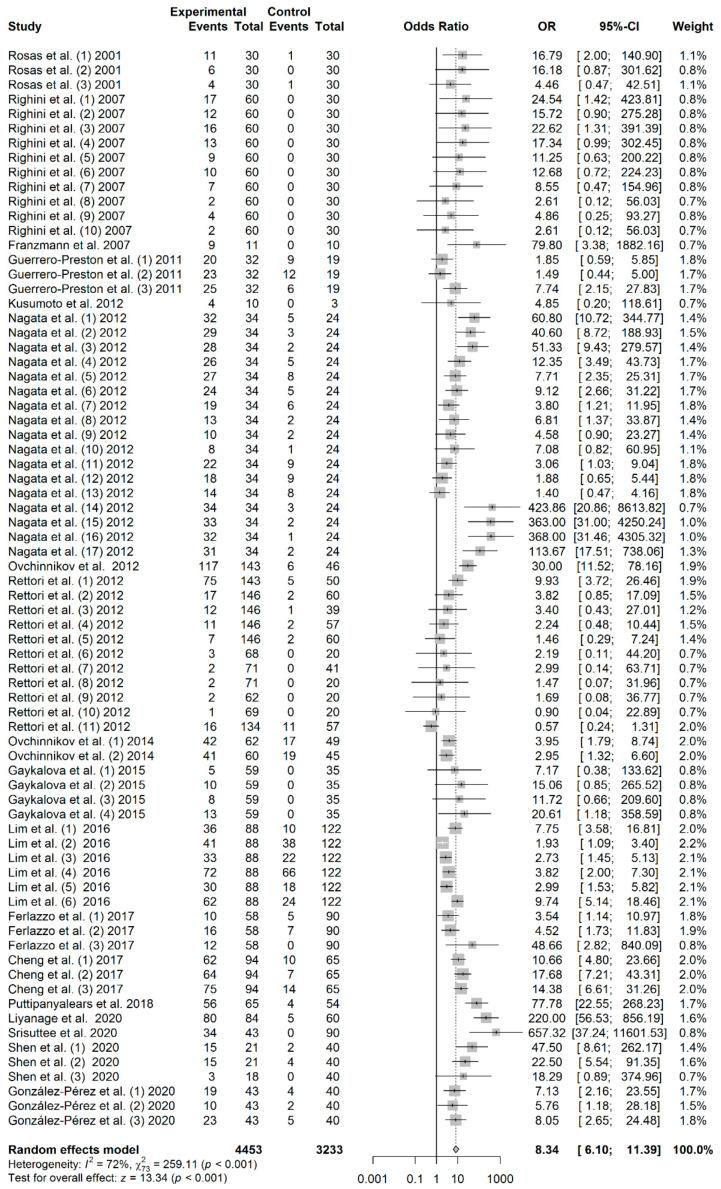
Forest plot for the association between salivary DNA promoter hypermethylation and the HNC risk. The squares represent the ORs for individual studies. Bars represent the 95% CIs. The center of the diamond represents the summary effect size.

A total of 7686 subjects, consisting of 4453 patients and 3233 controls, were included in this meta-analysis. As shown in Figure 1, the pooled analysis revealed a significant association between salivary DNA promoter hypermethylation and HNC with an OR of 8.34 (95% CI = 6.10–11.39; *p* < 0.01). A random-effects model was used because heterogeneity among the 18 studies (I^2^ = 72%) was identified. The shape of the Begg’s funnel plot did not reveal potential asymmetry (*p* = 0.271), although publication bias was detected by Egger’s test (*p* = 0.002) (Figure S1). 

### 3.4. Meta-Regression and Subgroup Analysis

Due to the presence of significant heterogeneity in the overall analysis, meta-regression and subgroup analysis were performed in order to reveal potential sources. The outcomes of meta-regression analysis showed that sample type (*p* = 0.128), sample size (*p* = 0.349), and DNA methylation method (*p* = 0.275) were not significant sources of heterogeneity. However, anatomic tumor location (*p* = 0.002) and gene profiling (*p* < 0.001) were, in fact, potential sources of heterogeneity in this study (Table S1—see Part I) [13]. As shown in Table S2, significant heterogeneity was found in all subgroups. With respect to sample type-based subgroup analysis, a significant association between promoter hypermethylation and HNC was found in oral rinse samples (OR: 9.42; 95% CI = 6.30–14.08) and saliva samples (OR: 6.33; 95% CI = 3.90–10.27). In tumor-based subgroup analysis, methylation rates were higher in specific head and neck locations compared to studies that made no differentiation. The pooled OR for oropharyngeal cancer was 13.26 (95% CI = 3.17–5.42) and for oral cancer was 13.07 (95% CI = 8.19–20.88), while for HNC it was 5.78 (95% CI = 3.86–8.67). A significant association between salivary promoter methylation and HNC was found by both MSP (OR: 9.06; 95% CI = 6.30–13.03) and qMSP (OR: 6.81; 95% CI = 3.70–12.54) techniques. With respect to the subgroups categorized by sample size, a significant association was found between salivary promoter methylation and HNC in studies with N < 100 (OR: 9.58; 95% CI = 6.44–14.27) and N > 100 (OR: 8.34; 95% CI = 6.10–11.39). In subgroup analysis based on the gene-profiling approach, salivary promoter hypermethylated gene panels had a significantly higher association to HNC risk (OR: 36.79; 95% CI = 16.81–81.32) than hypermethylated single genes (OR: 6.02; 95% CI = 4.46–8.13). 

### 3.5. Association between p16 Promoter Hypermethylation and HNC Risk

**Figure 2:**
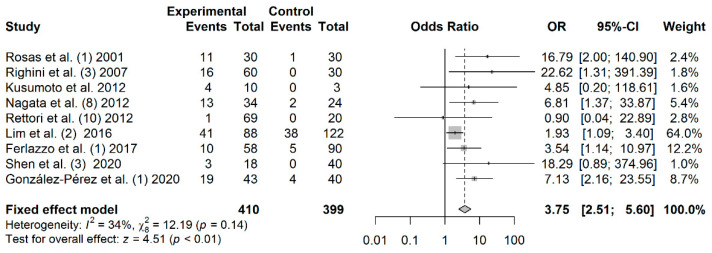
Forest plot for the association between *p16* promoter hypermethylation and HNC risk. The squares represent the ORs for individual studies. Bars represent the 95% CIs. The center of the diamond represents the summary effect size.

A total of 410 cases and 399 controls from 9 studies were included to estimate the effect of *p16* promoter hypermethylation on HNC risk. As shown in Figure 2, a significant association was found between salivary *p16* promoter hypermethylation and HNC risk (OR: 3.75; 95% CI = 2.51–5.60). The shape of the Begg’s funnel plot did not reveal potential asymmetry (*p* = 1), although publication bias was detected by Egger’s test *(p* = 0.040) (Figure S2).

### 3.6. Association between MGMT Promoter Hypermethylation and HNC Risk

**Figure 3:**
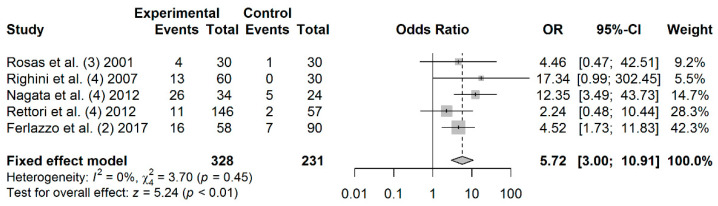
Forest plot for the association between *MGMT* promoter hypermethylation and HNC risk. The squares represent the ORs for individual studies. Bars represent the 95% CIs. The center of the diamond represents the summary effect size.

A total of 328 cases and 231 controls from 5 studies were included to estimate the effect of *MGMT* promoter hypermethylation on HNC risk. As shown in Figure 3, salivary *MGMT* promoter hypermethylation was associated with an increased HNC risk (OR: 5.72; 95% CI = 3.00–10.91). Visual analysis of the funnel plot revealed a symmetrical distribution of the studies (Egger’s test, *p* = 0.767; Begg’s test, *p* = 0.624), indicating no evidence of publication bias (Figure S3). 

### 3.7. Association between DAPK Promoter Hypermethylation and HNC Risk

**Figure 4:**
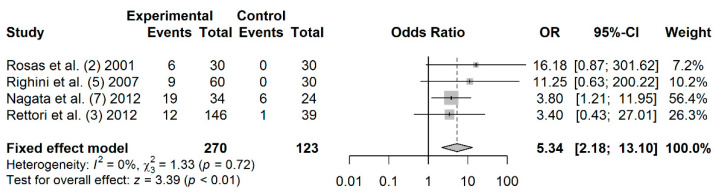
Forest plot for the association between *DAPK* promoter hypermethylation and HNC risk. The squares represent the ORs for individual studies. Bars represent the 95% CIs. The center of the diamond represents the summary effect size.

A total of 270 cases and 123 controls from 4 studies were included to estimate the effect of *DAPK* promoter hypermethylation on HNC risk. As shown in Figure 4, the rate of salivary *DAPK* promoter hypermethylation was significantly higher in HNC patients compared to controls (OR: 5.34; 95% CI = 2.18–13.10). Visual examination of the funnel plot revealed a symmetrical distribution of the studies (Begg’s test, *p* = 0.041; Egger’s test, *p* = 0.187;), indicating no evidence of publication bias (Figure S4).

### 3.8. Association between TIMP3 Promoter Hypermethylation and HNC Risk

**Figure 5:**
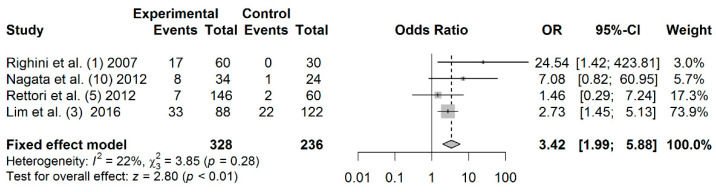
Forest plot for the association between TIMP3 promoter hypermethylation and HNC risk. The squares represent the ORs for individual studies. Bars represent the 95% CIs. The center of the diamond represents the summary effect size.

A total of 328 cases and 236 controls from 4 studies were included to estimate the effect of *TIMP3* promoter hypermethylation on HNC risk. As shown in Figure 5, a significant association was found between salivary *TIMP3* promoter hypermethylation and HNC risk (OR: 3.42; 95% CI = 1.99–5.88). Visual inspection of the funnel plot revealed a symmetrical distribution of the studies (Begg’s test, *p* = 0.174; Egger’s test, *p* = 0.419), indicating no evidence of publication bias (Figure S5). 

### 3.9. Association between RASSF1A Promoter Hypermethylation and HNC Risk

**Figure 6:**
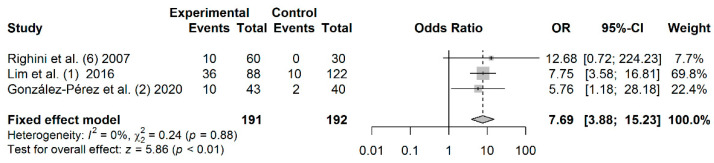
Forest plot for the association between *RASSF1A* promoter hypermethylation and the HNC risk. The squares represent the ORs for individual studies. Bars represent the 95% CIs. The center of the diamond represents the summary effect size.

A total of 191 cases and 192 controls from 3 studies were included to estimate the effect of *RASSF1A* promoter hypermethylation on HNC risk. As shown in Figure 6, salivary *RASSF1A* promoter hypermethylation was associated with an increased HNC risk (OR: 7.69; 95% CI = 3.88–15.23). Visual examination of the funnel plot revealed a symmetrical distribution of the studies (Begg’s test, *p* = 0.601; Egger’s test, *p* = 0.858), indicating no evidence of publication bias (Figure S6). 

### 3.10. Association between APC Promoter Hypermethylation and HNC Risk

**Figure 7:**
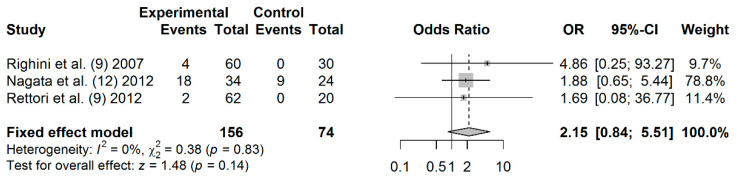
Forest plot for the association between *APC* promoter hypermethylation and HNC risk. The squares represent the ORs for individual studies. Bars represent the 95% CIs. The center of the diamond represents the summary effect size.

A total of 156 cases and 74 controls from 3 studies were included to estimate the effect of *APC* promoter hypermethylation on HNC risk. As shown in Figure 7, salivary *APC* promoter hypermethylation was not significantly associated with HNC (OR: 2.15; 95% CI = 0.84–5.51). Visual examination of the funnel plot revealed no potential asymmetry (Begg’s test, *p* = 0.601; Egger’s test, *p* = 0.609), indicating no evidence of publication bias (Figure S7).

## 4. Discussion

Aberrant DNA hypermethylation has been recognized as an important epigenetic mechanism involved in head and neck carcinogenesis [1], suggesting its potential as a biomarker for evaluating cancer risk. Although prior studies have focused on the detection of promoter DNA hypermethylation in saliva from HNC patients [10,17], the evidence of a direct relationship is unclear and findings have been inconsistent. 

To the best of our knowledge, this is the first meta-analysis evaluating the contribution of salivary promoter hypermethylation to HNC risk. The present comprehensive analysis included 18 studies comprising 4453 patients and 3233 controls. Overall, our results indicate that salivary promoter hypermethylation was significantly associated with an 8.34-fold increase in HNC risk. 

As significant heterogeneity was observed among studies, meta-regression and subgroup analyses were carried out based on anatomic tumor location, sample type, sample size, DNA methylation method, and methylation gene profiling. The stratified analysis revealed that salivary DNA hypermethylation was associated with HNC risk in all subgroups. The association between salivary DNA promoter hypermethylation and HNC risk was stronger in oral rinses compared to saliva. This could be explained by the higher methylation proportion of oral exfoliated cells in oral rinse compared to saliva samples. Subgroup analysis of anatomic tumor location showed that the OR was higher in oral cancer and oropharyngeal cancer than overall HNC. These findings could be explained by the direct contact of saliva samples with tumors located in the oral cavity and oropharynx, which could result in an increased number of exfoliated tumoral cells during sample collection. Based on the methylation detection method subgroup, the frequency of salivary DNA promoter methylation was higher in MSP than in qMSP. This may be because MSP was the most commonly used technique for detecting aberrant DNA methylation in saliva samples (11 studies). In addition, the qualitative nature and lower specificity of MSP could lead to an overestimation of methylation data compared to qMSP methods [18]. However, quantitative approaches, such as qMSP or pyrosequencing, have shown better sensitivity than MSP [19]. With respect to sample size, a similar significant association was found between *n* < 100 and *n* > 100 subgroups. On the other hand, the gene profiling subgroup revealed that HNC risk was clearly higher when aberrant gene-specific DNA methylation was analyzed using gene panels rather than single gene analysis. This suggests that multiple tumor suppressor genes are epigenetically silenced in HNC pathogenesis, and, therefore, gene methylation panels should be used to better identify HNC risk.

We also explored the association between gene-specific promoter DNA methylation and HNC risk by analyzing the methylation frequency of genes reported in at least three studies. Thus, promoter hypermethylation of *p16*, *DAPK*, *TIMP3, MGMT,* and *RASSF1A* was significantly higher in HNC patients compared to controls, suggesting that the methylation of these tumor suppressor genes may play an important role in head and neck carcinogenesis. The *p16* gene acts as a negative cell cycle regulator that prevents the inactivation of retinoblastoma (Rb) protein by inhibiting the cyclin-dependent kinases and, therefore, cell cycle progression at G1/S phase [20]. Hypermethylation of *p16* promoter has been reported as a frequent epigenetic event in oral carcinogenesis [21,22]. In the present meta-analysis, methylation of *p16* promoter was significantly associated with a 3.75-fold increase in HNC risk, which is consistent with the study by Shi et al. (OR: 3.37) based on tissue and liquid biopsy methylation data [23]. In line with this, a more recent meta-analysis comprising 67 case-control studies reported an OR of 6.72. However, subgroup analysis in this study based on sample type revealed that OR was much higher in saliva (OR: 12.45) and blood (OR: 16.40) than in tissue (OR: 6.40) [24]. Overall, these findings indicate that hypermethylation of *p16* gene promoter in saliva could be a predictive biomarker for HNC risk. The *MGMT* gene is involved in the repair of O6-methylguanine in DNA sequences originating from the carcinogenic effects of alkylating agents [25]. The inactivation of *MGMT* promoter by aberrant hypermethylation has been associated with an increased frequency of GC > AT transition mutations in *TP53* and in *KRAS* oncogene, contributing to carcinogenesis and tumor progression [26,27]. In fact, our meta-analysis showed that methylation of *MGMT* promoter leads to a 5.72-fold increase in HNC risk. *DAPK* plays a critical role in the apoptotic process triggered by interferon-gamma (IFN-γ), tumor necrosis factor (TNF)-alpha, Fas ligand, and detachment from extracellular matrix [28]. Hypermethylation of *DAPK* gene promoter is a frequent alteration in HNC [29,30]. The results of the present meta-analysis show that individuals with salivary hypermethylation of *DAPK* gene promoter had a 5.34-fold higher HNC risk. A previous meta-analysis also showed that the frequency of *DAPK* promoter methylation was significantly higher in HNC vs. control groups (OR: 6.72) [31]. The *TIMP3* gene is a tissue inhibitor of matrix metalloproteinases, which acts as a potential anticancer agent by inducing apoptosis and inhibiting proliferation, angiogenesis, and metastasis [32]. The methylation of *TIMP3* promoter has been associated with HNC [33,34]. Interestingly, our meta-analysis revealed a significant association between salivary *TIMP3* promoter methylation and HNC with an OR of 3.42. The *RASSF1A* gene prevents tumorigenesis through multiple cellular process, such as cell cycle arrest, migration, microtubular stabilization, and apoptosis promotion [35]. Epigenetic inactivation of *RASSF1A* by hypermethylation has been observed in various cancers, including HNC [36]. Our data showed that methylation of *RASSF1A* promoter led to a 7.69-fold increase in HNC risk compared to the control group. In a previous study, Meng et al. evaluated the methylation prevalence of *RASSF1A* between cancerous tissues and controls, finding a significant association (OR: 2.93) between aberrant methylation of *RASSF1A* and HNC [37]. The *APC* gene acts as a negative regulator in the Wnt/beta-catenin signaling pathway and its dysfunction leads to increased β-catenin transcriptional activity, promoting the activation of downstream targets involved in tumorigenesis, such as cyclin D1 and Myc [38]. Hypermethylation of the *APC* promoter has been reported as a mechanism for *APC*-gene inactivation in oral carcinogenesis [39]. Our study did not reveal a significant association between salivary *APC* promoter hypermethylation and HNC, which could be explained by the low *APC*-gene methylation rates detected in saliva from HNC patients. Until now, few studies have reported *APC* hypermethylation in saliva from HNC patients [40,41,42]; however, this epigenetic alteration has been frequently observed in head and neck tumors [29,39,43,44]. It is important to note that hypermethylation of *p16*, *DAPK*, *TIMP3, MGMT*, and *RASSF1A* plays an important role in the carcinogenesis of various tumors, such as lung, breast, colorectal, renal, or gastric [45,46,47,48,49,50]. In line with this, several studies have focused on the association of cancer risk with the hypermethylation of these tumor suppressor genes [51,52,53,54,55], which highlights its potential for early diagnosis of the disease.

The present study has several strengths. It is the first meta-analysis highlighting the association between salivary DNA promoter hypermethylation and HNC. It explores the magnitude of the association both overall and by specific hypermethylated gene. In addition, it involved a comprehensive literature review without language restrictions. However, our study is not exempt from limitations. Firstly, all included research involved case-control retrospective studies, which could lead to selection bias. Some bias could also stem from the fact that cases and controls were not matched for demographic variables, such as age, sex, and lifestyle habits. Secondly, significant heterogeneity was found among studies. Despite performing subgroup analysis by anatomic tumor location, sample type, sample size, DNA methylation method, and methylation gene profiling, we were unable to elucidate the potential sources of this heterogeneity. Further subgroup analysis was hindered by the lack of original data regarding lifestyle habits or ethnicity. Thirdly, the association of salivary DNA promoter hypermethylation and clinicopathological variables (i.e., TNM stage, histological grade) was not explored due to insufficient data. Therefore, well-designed prospective clinical studies with large sample sizes are necessary to validate the results of this meta-analysis.

## 5. Conclusions

Overall, the findings from this meta-analysis showed that salivary DNA promoter hypermethylation was associated with HNC risk. Salivary hypermethylation of *p16*, *MGMT*, *DAPK*, *TIMP3,* and *RASSF1A* showed an important role in HNC development. Thus, saliva could be used as a potential source of epigenetic biomarkers for predicting HNC. The development of HNC screening programs based on the combination of these 5-methylated genes in saliva could be useful for identifying high-risk patients and for detecting cancer before the occurrence of initial clinical symptoms. The clinical implementation of this salivary panel would represent the beginning of precision medicine for HNC. To attain this, prospective and multicenter studies should be carried out in order to validate the present results.

## Supplementary Materials

The following are available online at https://www.mdpi.com/article/10.3390/jpm11070606/s1, Figure S1: Funnel plot for studies (of 18 studies) on the association between salivary DNA hypermethylation and HNC, Figure S2: Funnel plot for studies (of 9 studies) on the association between salivary hypermethylation of *p16* gene promoter and HNC, Figure S3: Funnel plot for studies (of 5 studies) on the association between salivary hypermethylation of *MGMT* gene promoter and HNC, Figure S4: Funnel plot for studies (of 4 studies) on the association between salivary hypermethylation of *DAPK* gene promoter and HNC, Figure S5 Funnel plot for studies (of 4 studies) on the association between salivary hypermethylation of *TIMP3* gene promoter and HNC, Figure S6: Funnel plot for studies (of 3 studies) on the association between salivary hypermethylation of *RASSF1A* gene promoter and HNC, Figure S7: Funnel plot for studies (of 3 studies) on the association between salivary hypermethylation of *APC* gene promoter and HNC, Table S1: The Newcastle-Ottawa Scale (NOS) for assessing the quality of included studies, Table S2: Subgroup analysis of salivary DNA methylation for HNC detection based on different covariates.
