# Supplementary material for: Salivary DNA Methylation as an Epigenetic Biomarker for Head and Neck Cancer. Part II: A Cancer Risk Meta-Analysis
Source: J Pers Med. 2021 Jun 26;11(7):606. doi: 10.3390/jpm11070606 (PMC8304899; doi:10.3390/jpm11070606)
Supplement: Supplementary file 1 [file jpm-11-00606-s001.zip › jpm-1227624-supplementary.pdf]

Supplementary Material

Salivary DNA methylation as an epigenetic biomarker for head and neck cancer. Part II: A cancer risk meta-analysis

Óscar Rapado-González, Cristina Martínez-Reglero, Ángel Salgado-Barreira, María Arminda Santos, Rafael López-López, Ángel Díaz-Lagares and María Mercedes Suárez-Cunqueiro

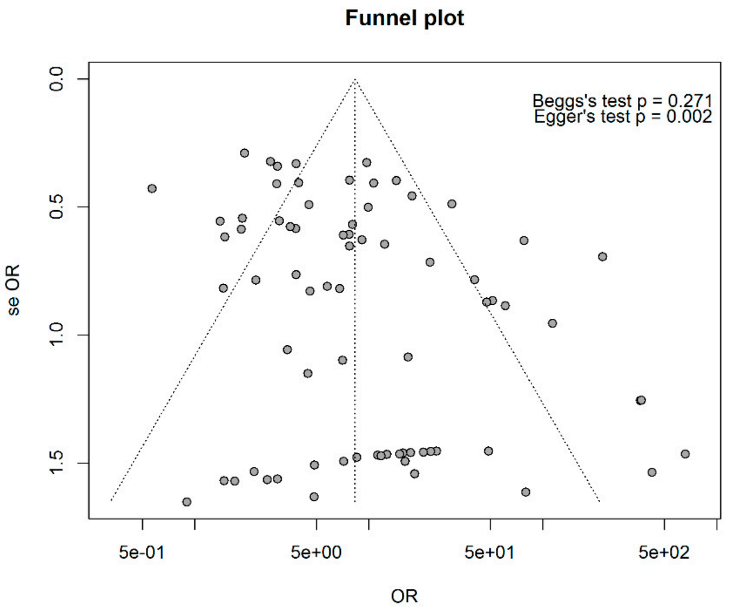

Figure S1. Funnel plot for studies (of 18 studies) on the association between salivary DNA hypermethylation and HNC. *Abbreviations:* HNC = head and neck cancer.

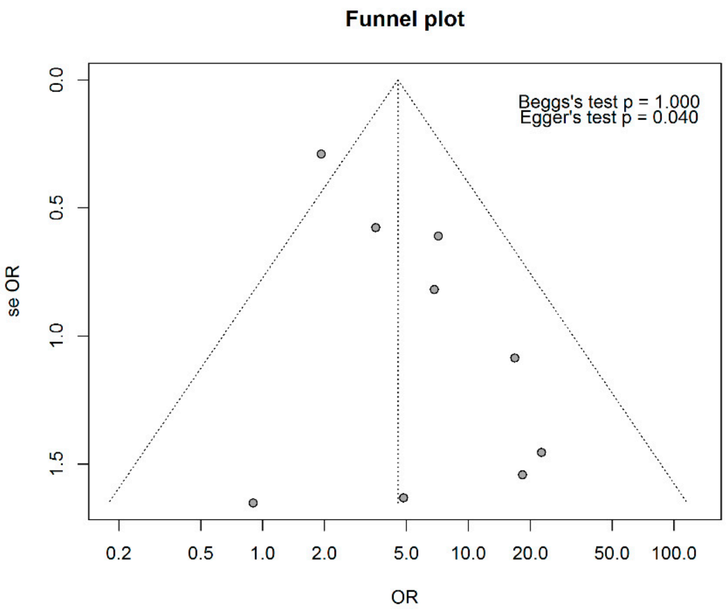

Figure S2. Funnel plot for studies (of 9 studies) on the association between salivary hypermethylation of *p16* gene promoter and HNC. *Abbreviations:* HNC = head and neck cancer.

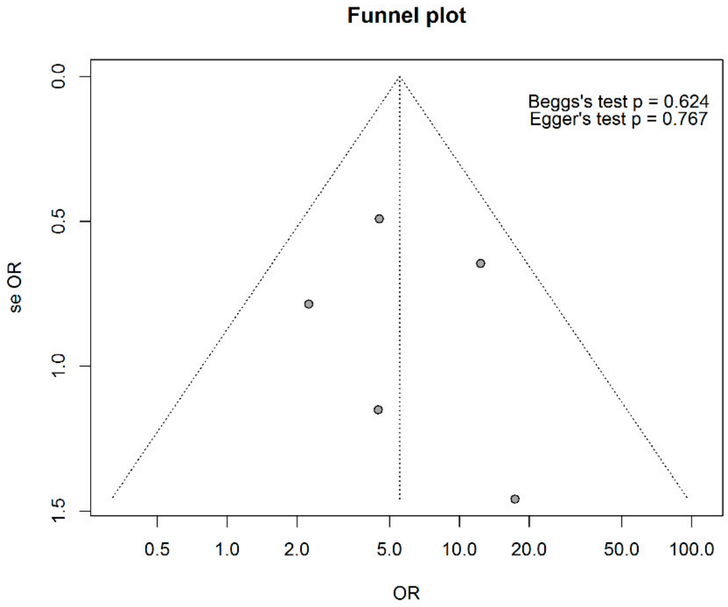

Figure S3. Funnel plot for studies (of 5 studies) on the association between salivary hypermethylation of *MGMT* gene promoter and HNC. *Abbreviations:* HNC = head and neck cancer.

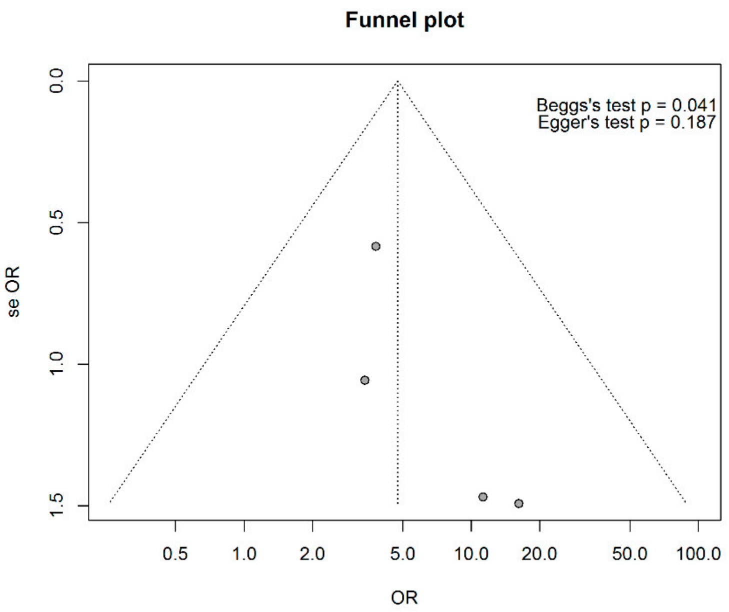

Figure S4. Funnel plot for studies (of 4 studies) on the association between salivary hypermethylation of *DAPK* gene promoter and HNC. *Abbreviations:* HNC = head and neck cancer.

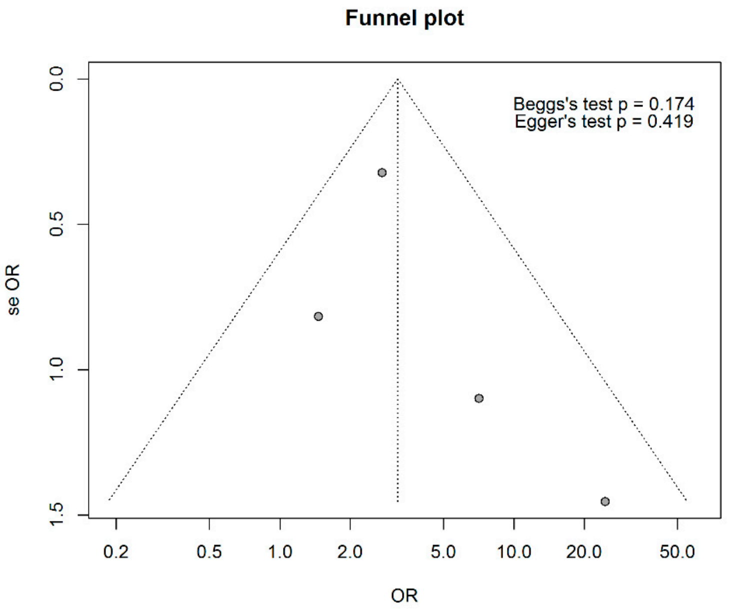

Figure S5. Funnel plot for studies (of 4 studies) on the association between salivary hypermethylation of *TIMP3* gene promoter and HNC. *Abbreviations:* HNC = head and neck cancer.

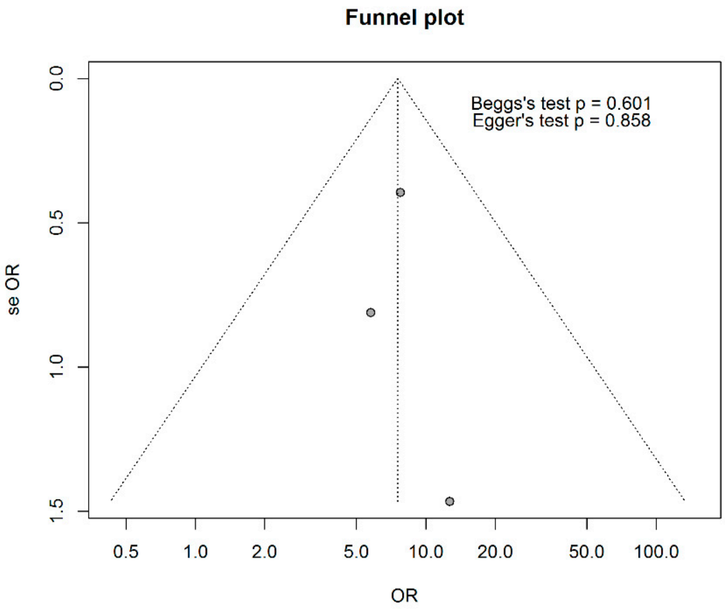

Figure S6. Funnel plot for studies (of 3 studies) on the association between salivary hypermethylation of *RASSF1A* gene promoter and HNC. *Abbreviations:* HNC = head and neck cancer.

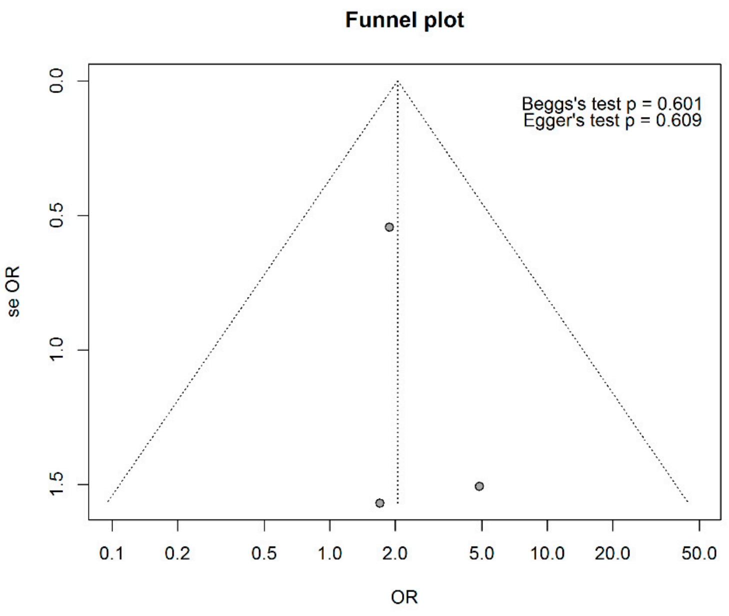

Figure S7. Funnel plot for studies (of 3 studies) on the association between salivary hypermethylation of *APC* gene promoter and HNC. *Abbreviations:* HNC = head and neck cancer.

Table S1. The Newcastle-Ottawa Scale (NOS) for assessing the quality of included studies.

**Study**	**Subject Selection (Max. 4★)**	**Study Comparability (Max. 2★)**	**Ascertainment of exposure (Max. 3★)**	**Final quality assessment**
Rosas et al.; 2001	★★★★	0	★★★	7
Righini et al.; 2007	★★★★	0	★★★	7
Franzmann et al.; 2007	★★	★★	★★★	7
Guerrero-Preston et al.; 2011	★★★★	0	★★★	7
Nagata et al.; 2012	★★★★	0	★★★	7
Ovchinnikov et al.; 2012	★★★★	0	★★★	7
Ksumoto et al.; 2012	★★★★	0	★★★	7
Rettori et al.; 2012	★★★★	0	★★★	7
Ovchinnikov et al.; 2014	★★★★	0	★★★	7
Gaykalova et al.; 2015	★★★★	0	★★★	7
Lim et al.; 2016	★★★★	0	★★★	7
Ferlazzo et al.; 2017	★★★★	★★	★★★	9
Cheng et al.; 2017	★★★★	0	★★★	7
Puttipanyalears et al.; 2018	★★★★	0	★★★	7
Liyanage et al.; 2020	★★★★	★★	★★★	9
Srisutte et al.; 2020	★★★★	★	★★★	8
Shen et al.; 2020	★★★★	0	★★★	7
González-Pérez et al.; 2020	★★★★	★	★★★	8

Note: "★" means one point

Table S2. Subgroup analysis of salivary DNA methylation for HNC detection based on different covariates.

Abbreviations: HNC = head and neck cancer; OC = oral cancer; OPC = oropharyngeal cancer; MSP = methylation-specific polymerase chain reaction; qMSP = quantitative-MSP.

**Subgroups**	**No of study units**	**Sample size (cases/controls)**	**Heterogeneity**		**Model**	**Effect size**	
			**I^2(%)**	**$P_{Q\text{-}test}$**		**OR (95% CI)**	***P-value***
**Sample type**							
Saliva	16		79	<0.01	R	6.33 (3.90–10.27)	<0.01
Oral Rinse	58		68	<0.01		9.42 (6.30–14.08)	<0.01
**Samples size**							
N>100	25		84	<0.01	R	8.34 (6.10–11.39)	<0.01
N<100	49		53	<0.01		9.58 (6.44–14.27)	<0.01
**Tumor Location**							
HNC	43		70	<0.01	R	5.78 (3.86–8.67)	<0.01
OC	33		73	<0.01		13.07 (8.19–20.88)	<0.01
OPC	8		80	<0.01		13.26 (3.17–5.42)	<0.01
**Technique**							
MSP	48		70	<0.01	R	9.06 (6.30–13.03)	<0.01
qMSP	26		75	<0.01		6.81 (3.70–12.54)	<0.01
**Gene profiling**							
Single gene	62		63	<0.01	R	6.02 (4.46–8.13)	<0.01
Combination gene	12		74	<0.01		36.79 (16.81–81.32)	<0.01
